# Supplementary material for: Identification of photoperiod-induced specific miRNAs in the adrenal glands of Sunite sheep (Ovis aries)
Source: Front Vet Sci. 2022 Jul 22;9:888207. doi: 10.3389/fvets.2022.888207 (PMC9354845; doi:10.3389/fvets.2022.888207)
Supplement: Supplementary file 1 [file Data_Sheet_1.ZIP › Supplementary file/miRDeep2 file.zip!/miRDeep2 file/Know_Structure/LP42A3/oar-mir-3957.pdf]

miRBase precursor : oar-mir-3957
Total read count : 158
oar-miR-3957-5p read count : 132
oar-miR-3957-3p read count : 25
remaining reads : 1

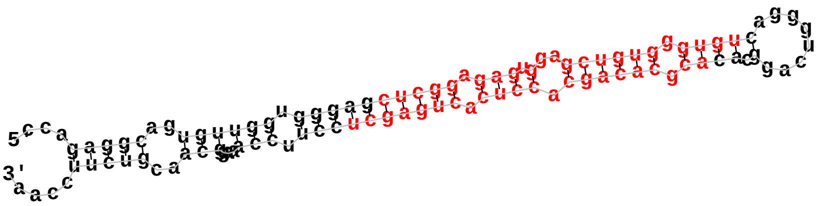

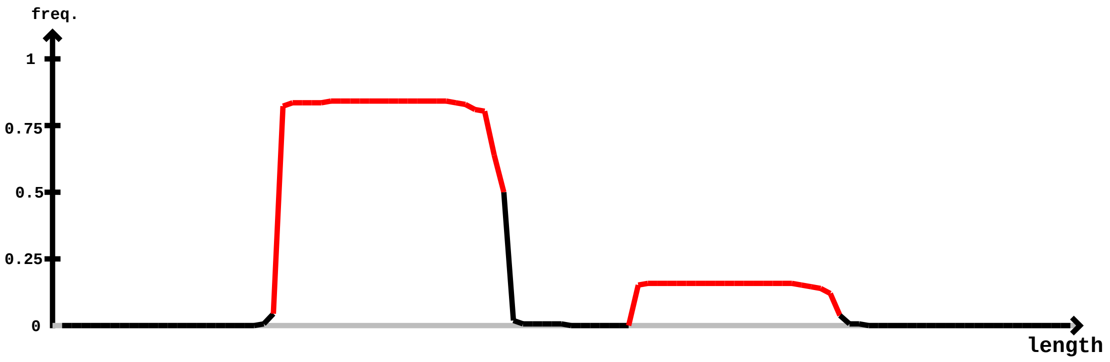


```
                                    oar-miR-3957-5p
                                                                        oar-miR-3957-3p
5'- ccagaggcaguguuggugggagcucggagaguggagcugugggugucagggucaggcacacgcacagcaccucacugagcuccuuccaaaggcaacgucuuccaa   -3'    exp
    ...(((((..((((((.((((((((((.(((.(..((((((.(((((........).)))).)))))).)))).)))))))))).)))...)))..)))))....   reads    mm    sample
    ....................agcucggagaguggagcuguggg..............................................................   1        0     seq
    .....................gcucggagaguggagcugu.................................................................   1        0     seq
    .....................gcucggagaguggagcugugggu.............................................................   3        0     seq
    .....................gcucggagaguggagcuguggug............................................................   1        0     seq
    .....................gcucggagaguggagcugugggugu...........................................................   1        0     seq
    ......................cucggagaguggagcugug................................................................   1        0     seq
    ......................cucggagaguggagcugugg...............................................................   3        0     seq
    ......................cucggagaguggagcugugggu.............................................................   23       0     seq
    ......................cucggagaguggagcuguggug............................................................   21       0     seq
    ......................cucggagaguggagcugugggugu...........................................................   73       0     seq
    ......................cucggagaguggagcugugggugucc..........................................................   2        0     seq
    .......................ucggagaguggagcugugggugu...........................................................   2        0     seq
    ...........................agaguggagcugugggugucagggu.....................................................   1        0     seq
    ...........................................................acgcacagcaccucacu.............................   1        0     seq
    ...........................................................acgcacagcaccucacug............................   1        0     seq
    ...........................................................acgcacagcaccucacuga...........................   1        0     seq
    ...........................................................acgcacagcaccucacugag..........................   3        0     seq
    ...........................................................acgcacagcaccucacugagc.........................   13       0     seq
    ...........................................................acgcacagcaccucacugagcu........................   5        0     seq
    ............................................................cgcacagcaccucacugagcucc......................   1        0     seq
```
